# Relationship between High Temperature and Formation of Chalkiness and Their Effects on Quality of Rice

**DOI:** 10.1155/2018/1653721

**Published:** 2018-04-01

**Authors:** A. Y. M. Nevame, R. M. Emon, M. A. Malek, M. M. Hasan, Md. Amirul Alam, Farrah Melissa Muharam, Farzad Aslani, M. Y. Rafii, M. R. Ismail

**Affiliations:** ^1^State Key Laboratory of Rice Biology, China National Rice Research Institute, Hangzhou 310006, China; ^2^Bangladesh Institute of Nuclear Agriculture, BAU Campus, Mymensingh 2202, Bangladesh; ^3^Institute of Tropical Agriculture and Food Security, Universiti Putra Malaysia (UPM), 43400 Serdang, Selangor, Malaysia; ^4^School of Agriculture Science and Biotechnology, Faculty of Bioresources and Food Industry, Universiti Sultan Zainal Abidin, Besut Campus, 22200 Besut, Terengganu, Malaysia; ^5^Laboratory of Science and Technology, Institute of Plantation Studies, Universiti Putra Malaysia (UPM), 43400 Serdang, Selangor, Malaysia; ^6^Department of Crop Science, Faculty of Agriculture, Universiti Putra Malaysia (UPM), 43400 Serdang, Selangor, Malaysia

## Abstract

Occurrence of chalkiness in rice is attributed to genetic and environmental factors, especially high temperature (HT). The HT induces heat stress, which in turn compromises many grain qualities, especially transparency. Chalkiness in rice is commonly studied together with other quality traits such as amylose content, gel consistency, and protein storage. In addition to the fundamental QTLs, some other QTLs have been identified which accelerate chalkiness occurrence under HT condition. In this review, some of the relatively stable chalkiness, amylose content, and gel consistency related QTLs have been presented well. Genetically, HT effect on chalkiness is explained by the location of certain chalkiness gene in the vicinity of high-temperature-responsive genes. With regard to stable QTL distribution and availability of potential material resources, there is still feasibility to find out novel stable QTLs related to chalkiness under HT condition. A better understanding of those achievements is essential to develop new rice varieties with a reduced chalky grain percentage. Therefore, we propose the pyramiding of relatively stable and nonallelic QTLs controlling low chalkiness endosperm into adaptable rice varieties as pragmatic approach to mitigate HT effect.

## 1. Introduction

Rice (*Oryza sativa* L.) is an important cereal crop worldwide. It is the staple food for more than half of the world's population [1]. Development of quality rice varieties has been the principle objective throughout the history of plant breeding. A priority issue in many rice-producing areas of the world along with improvements in the standard of living, the demand for superior grain quality is increasing day by day [2–4]. Rice grain quality is a complex characteristic including many components such as appearance, cooking qualities, and eating qualities. Among these properties, consumers often pay most attention to the appearance [5, 6]. Several genetic and environmental factors were shown to influence rice grain appearance, which is usually evaluated as percentage of grains with chalkiness (PGWC) [7].

Chalkiness is reported to occur not only under the complex genetic network regulation [8] but also under the effects of environmental factors, especially heat stress [9, 10]. Generally, different rice cultivars show different PGWC [11]. Studies showed the rice grain quality traits to be polygenic, while many quantitative trait loci (QTLs) associated with grain quality have been identified in rice [12–14]. Although several QTLs of rice grain appearance quality were detected, it is still difficult to fully use these QTLs in rice gene pyramiding breeding. Furthermore, the genetic basis of appearance quality traits is explained by many QTLs with various effects in different environments [15]. In addition, their phenotypic expression is generally affected by pleiotropic effects of genes for nontarget traits [16].

Frequently heat stress occurs concurrently with salinity and drought stresses, which disturb cellular homeostasis and hamper plant growth and developmental process [17–20]. Heat stress was identified as a major inducible factor of chalkiness formation in rice. Expression of heat shock proteins (HSPs) was mentioned as basic metabolic defense in response to heat stress in the plants and other organisms [21, 22] However, ectopic expression of a single heat stress tolerance gene could not completely resolve harmful stress effects on the plant due to the complexity of this trait [21]. Scientists are still concerned about this problem; therefore, they reiterated that efforts are needed to overcome heat stress effect [23, 24]. In this review, the mechanism/pathways of heat stress to cause chalkiness formation in grain are presented. Moreover, genetic approach towards offering a solution for reducing stress effect is also illustrated in Perspectives: Pyramiding of Nonallelic Stable QTLs Controlling Low Chalkiness Formation under HT Condition in Rice.

## 2. Rice Grain Quality

**Figure 1:**
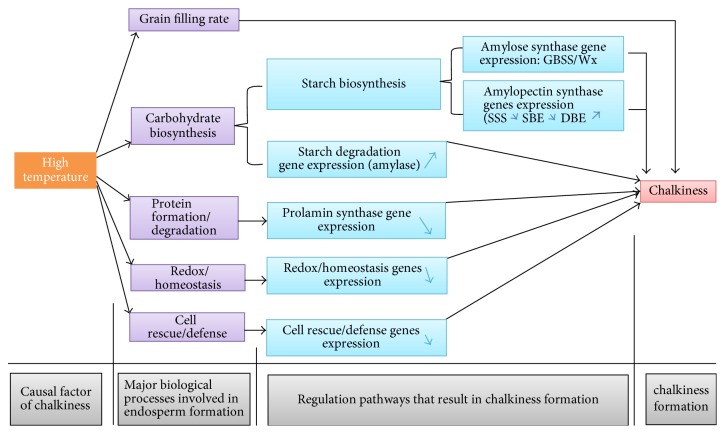
Principal environmental causal factors and the mechanism pathways of the chalkiness formation in rice. Up arrows designate upregulation of the starch synthase genes and down arrows designate downregulation of synthase starch genes. Yellow box constituted the causal factor of the chalkiness formation, the purple boxes are the major physiological processes involved in endosperm formation, the light blue represents the regulatory pathways resulting in chalkiness formation, and red box is chalkiness formation. Source: Chen et al. [25].

HT affects both grain yield and quality in rice. Rice quality is an important character that influences its consumption rate, thus enhancing its production. Therefore, the improvement of grain quality becomes a second major concern in rice breeding program after yield. In rice, HT has been reported to reduce grain seed size and yield [29] while also affecting endosperm quality [30]. Rice consumers recognize that different areas produce differing qualities of the rice grain [31–37]. Grain quality is a complex character that is determined by milling recovery, appearance, cooking, eating, and nutritional qualities [38]. Genetic and environmental factors have been reported to influence physical-biochemical properties of grain endosperm [38] as illustrated in this review (Figure 1(a)). Among the grain quality traits, endosperm appearance (chalkiness) remained a principal factor that determines market value of the rice grain. These quality traits are interrelated in that any change in appearance is likely to affect the milling and cooking properties.

## 3. Chalkiness in Rice

Chalkiness is an undesirable trait that negatively affects milling, cooking, eating, and grain appearance and represents a major problem in many rice-producing areas of the world [10, 39–41]. Rice consumers and producers express lack of interest in rice varieties with high degree of chalky grain. For example, chalkiness was reported to have negative influence on farmers' preference for rice varieties cultivated in Ghana [42]. The percentage of chalkiness in rice grain is an index that determines the appearance quality. Chalky grains were found to contain a lower density of starch granules as compared to vitreous ones [43]. So, for improving the milling and cooking quality, rice endosperm should be free from chalkiness. Different types of chalkiness such as white core, white back, and white belly were reported to be present in the endosperm of rice grain [38].

### 3.1. Genetic Factors Responsible for Chalkiness Formation in Rice

Chalkiness is an endosperm related quality trait and its inheritance was reported to be controlled by triploid endosperm genotype, diploid maternal genotype, and any additional cytoplasm factors [44–46]. Currently, more than 84 QTLs were reported to be associated with different types of chalkiness in rice [11, 12, 38, 47–49]. Wan et al. [11] detected 22 QTLs for appearance quality traits in recombinant inbred lines and chromosome segment substitution lines. Zheng et al. [50] found a total of 10 genomic regions with one major QTL on chromosome 7 which had large effects on rice grain shape and milling quality traits. The same has been observed consistently in several related rice populations. Hao et al. [3] identified 10 QTLs responsible for rice grain quality and eight QTLs responsible for physicochemical traits with one major QTL on chromosome 8 responsible for the percentage of chalky rice grains in a population of chromosome segment substitution lines. For example,* Chalk5* was found as a major QTL encoding a vacuolar H+-translocating pyrophosphatase which determines chalkiness formation in rice [51].* OsPPDKB* and starch synthase* IIIa (SSIIIa)* genes were found to have pleiotropic effects on white-core endosperm [52–54] indicating the relevance of these genes on endosperm formation in rice. The gene* OsPPDKB *controls the carbon flow into starch and lipid biosynthesis during grain filling [52], while the gene* SSIIIa* interferes with amylopectin chain elongation [53]. Soluble starch synthase* (SSS)*, starch branching enzyme (SBE), and starch debranching enzyme* (DBE)* also coordinate their actions for starch biosynthesis (Figure 1(b)). All these genes may interact to produce endosperm; thus disturbance in one function among them could cause metabolic dysfunction and chalkiness formation in plant. However, most of these genetic factors genes/QTLs were not detected under heat stress condition. It is quite obvious to only attribute endosperm appearance to the genetic factors, since rice varieties exhibit different endosperm quality under normal rice growing condition. Zheng et al. [55] used transparent endosperm variety Koshihikari (popular* Japonica *rice variety cultivated in Japan) and opaque endosperm variety Kasalath (*indica *variety possessing poor eating and cooking quality) to detect a percentage of grains with chalkiness QTL (PGWC) in rice. Tan et al. [7] detected white-belly chalkiness related QTL in recombinant inbreed lines (RILs) population derived from rice varieties Zhenshan 97 and Minghui 63. Bian et al. [14] identified 2 QTLs on rice chromosome one, which were related to percentage of grains with chalkiness by using introgression lines (ILs) population derived from rice varieties Sasanishiki and Habataki. Some of relatively stable QTLs controlling endosperm quality (chalkiness or opacity) in rice are listed in Table 1. At the same time, we have presented some QTLs related to amylose content and gel consistency, which are other grain characteristics often evaluated concomitantly with chalkiness (Tables 2 and 3).

### 3.2. High Temperature Induces Heat Stress That Affects Grain Quality in Rice

The global surface temperature is estimated to have increased between 1.8°C and 4°C over the 21st century [56]. Therefore, during the 40th session of the IPCC held in Paris, global warming is expected to be limited to 2°C by 2015 [57]. The gradual increase of the global temperature is thought to induce heat stress and generate reactive oxygen species in the plant [58]. This oxidative stress causes changes in plant metabolism and further compromises the yield and grain quality. Heat stress is a metabolic dysfunction imposed by higher temperature to the plant organism via biochemical, molecular, and physiological means, which affects cellular and whole-plant developmental process, which results in low crop yield and quality production.

It has been documented that warmer weather during ripening reduces heading rice percentage, deteriorating grain appearance, and worsens palatability due to chalkiness in rice grains [47, 59, 60]. Tashiro and Wardlaw [61] observed that temperatures higher than 26°C could easily cause chalky appearance and a reduction in grain weight. Okada et al. [62] have established the model for the multiple effects of temperature and radiation during the rice ripening period. They reported that an increase in temperature has a negative effect on rice grain quality, whereas an increase in radiation has a positive effect. In addition, these authors indicated that at temperature less than 21°C, most rice quality showed a high percentage of first-grade rice across the range of radiation, but at each temperature higher than 21°C, the decline of rice quality at lower radiation levels becomes increasingly pronounced. In conclusion, Okada et al. [62] stated that the sensitivity of rice quality to insufficient radiation increases as temperature increases. Tsukimori [63] reported that chalky grains increase sharply in number when the mean daily minimum temperature for 20 days after heading exceeds 23°C. Lanning et al. [9] have also confirmed the negative influence of night temperature on grain quality. Lyman et al. [64] reported that an increase of 1°C in average growing season temperature affects significantly the chalky and broken kernels rate in rice. High temperatures during grain filling process affect starch and protein storage, which result in a loosely parked starch granule deposition and thus increase chalky grains formation (Figure 1(b)). For example, Zakaria et al. [65] mentioned that higher temperature reduces the amount of large mature amyloplasts in the endosperm and increases the number of small immature ones. Yamakawa et al. [26] indicated that high temperature impairs grain filling by inhibiting the deposition of storage materials such as starch and protein in rice. This statement was supported by Gao et al. [66] who revealed that heat stress is responsible for the variation of amylose and protein contents in rice. So, it is understandable that heat stress reduces grain filling, which results in grain quality damage.

Morita and Nakano [67] have also reported that the occurrence of chalky grains under high temperatures is attributable mainly to the inhibition of starch accumulation. Moreover, these authors pointed out that the daily mean temperatures exceeding 26°C during the grain-filling period have caused chalkiness in the grains of* Japonica* cultivars. Lur et al. [68] described that cumulative temperature above 26°C within 15 days after heading can be regarded as an index of the extent of chalky grains. Wakamatsu et al. [69, 70] not only revealed that the ratios of white-back (WB) and basal-white (BW) grains increased when the average temperature during ripening exceeds 27°C but also noted the great variation in chalky grains ratio among* Japonica* rice cultivars under high-temperature conditions. Tsubone et al. [71] and Wakamatsu et al. [69, 70] indicated that the ratio of WB and BW grains increases if average air temperature during ripening exceeds 27°C. Wakamatsu et al. [72] reported that shading treatment mitigated the occurrence of WB in rice. Wada et al. [73] have detected some chalkiness related QTLs when air temperature exceeds 27°C (Table 4).

Afterwards, Liu et al. [74] indicated that heat stress affects the principal biological functions of grain endosperm formation such as carbohydrate synthesis, proteins formation/degradation, redox homeostasis, and cell defense (Figure 1(b))

**Figure 2:**
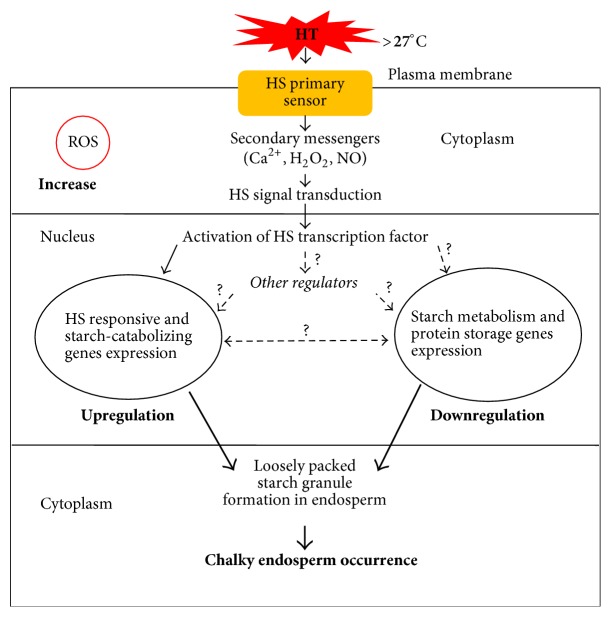
Heat stress affects HS responsive and starch-metabolizing genes. HS: heat shock. Arrows in bold indicate the mechanism pathways described by Yamakawa et al. [26, 27]. Dashed arrows are unclear regulation pathways or genetic interaction, which may be responsible for the chalkiness formation. Sample arrows represent HS effects transduction that leads to expression of HS protein genes expression [28]. ROS: reactive oxygen species.

Yamakawa et al. [26, 27] have conducted a transcriptome analysis and elucidated the molecular mechanism responsible for grain chalkiness and high-temperature-responsive genes. They observed that expression of genes controlling synthesis of starch and storage proteins was decreased by high temperature, while the expression of the genes related to starch decomposition and heat stress response were increased (Figure 2). To elucidate, Yamakawa et al. [27] have compared the chromosomal location of certain high-temperature-responsive genes to that of grain chalkiness QTLs. These authors found that some high-temperature-responsive genes are located in the vicinity of QTLs determining grain chalkiness. Thus, Yamakawa et al. [27] have localized several starch/carbohydrate-metabolizing enzyme genes on chromosome 8, especially* AGPS2 (ADP-glucose pyrophosphorylase 2)* between RM3181 and RM3689, which covered inner region of HT-sensitive QTL*qWB8* controlling chalkiness (white-back) in rice. Taken together, it appears that endosperm formation is a result of interaction between environment and genetic factors. For that reason, Lyman et al. [64] described HT as an important factor that determines the quantity and market value of milled rice. Heat stress regulates starch synthase enzymes activities in the plant. The effect of drought and temperature on expression of starch biosynthesis-related genes, such as* GBSS*,* SBE*s, and* BEIIb*, and amylases can directly or indirectly influence chalkiness formation in rice as mentioned by Chen et al. [25]. It can be preceded by reduction in activity of* GBSS*,* SBE*s, and* BEIIb*, while that of amylases increases [26, 75–78]. Heat stress was found to decrease prolamin content in rice due to the reduction in expression of its encoded genes,* Pro1* and* Pro2* [26, 38, 79]. It is clear that HT is one of the principal factors that induce chalkiness.

But, in order to mitigate effects of heat stress, plants need to restore metabolic damage by deploying appropriate metabolic pathways against oxidative stress. In this case, plants activate stress-responsive genes and generate antioxidant metabolites or heat shock protein (*HSP*) to reduce the harmful effects of heat [28, 80]. The* HSP*s were grouped into five families based on their molecular weights,* HSP100s, HSP90s, HSP70s, HSP60s, *and* sHSPs*, which have different degrees of performance across rice varieties [81]. Due to variant allelic interaction between living organisms, plants are different in terms of response to abiotic and biotic stress. While heat stress acts in complex manner, which affects simultaneously different metabolic pathways in plants, they need multiple responsive factors to restore damage caused by stress. Therefore, manipulation of multiple genes to overcome stress effect could be more reliable. Breeding efforts should then be directed to enhance metabolic defenses in plants in order to prevent damage caused by heat stress which eventually hampers the quality of rice produced.

### 3.3. QTLs Associated with Chalkiness under HT Condition

In previous studies, some chalkiness induced QTLs were screened out under high temperature condition in rice. These QTLs accelerate formation of chalkiness under HT condition. In this case, Kobayashi et al. [47] detected QTLs for white-back chalkiness on rice chromosomes 3, 4, and 6 by using recombinant inbred lines (RILs) derived from a cross between two strains of rice, heat stress-tolerant strain (Koshijiwase) and heat stress-sensitive strain (Chiyonishiki). In addition, Tabata et al. [60] identified QTLs for white-back chalkiness on chromosomes 1, 2, and 8 in RILs populations derived from a cross between heat stress-tolerant variety (Hanaechizen) and heat stress-sensitive variety (Niigatawase). Wada et al. [73] have identified 6 QTLs for white-back grains and basal-white grains on chromosomes 1, 2, 3, 8, and 12 under heat stress condition between two strains of rice: heat-tolerant variety (Chikushi 52) and heat stress-sensitive variety (Tsukushiroman). The QTL allele* qWB8* from Chikushi 52 variety was indicated to be important for improving grain quality under heat stress conditions [73]. Some stable HT induced chalkiness related QTLs are also mentioned in this review (Table 4). Moreover, various thermotolerant QTLs that were not directly related to chalkiness have been also identified in rice [30, 82–84]. For example, 4 QTLs that maintained proper rice amylose content at high temperature were detected from* Indica* rice variety 9311 (Table 2). Moreover, these QTLs were supposed to be essential for breeding heat-stable grain in rice [84]. These QTLs could constitute a potential genetic resource to improve this trait in rice.

### 3.4. Thermotolerant QTLs Unrelated to Chalkiness

A rice spotted leaf gene* Spl7* which encoded a transcription factor protein was mapped in Japanese rice cultivar Norin 8 as a heat stress-tolerant gene [85]. Heat tolerance QTL* (qHT4)* located on the chromosome region C1100-R1783 of the rice variety Kasalath was found to be stable over different environments [86]. The rice gene* OsHsfA2e*, a member of the heat stress transcription factors, when introduced into* Arabidopsis* line R04333 increased tolerance to heat stress. This gene was found to be useful in molecular breeding for stress tolerance improvement in crops [87]. High root activity and antioxidative defenses were reported as heat tolerance traits in* indica* rice cultivars Shuanggui and Huanghuazhan [30]. The reduced accumulation of ROS in leaf was observed in aus rice cultivar Nagina 22 under 40°C heat conditions [83]. Recently, major quantitative trait locus* (OgTT1)* with higher thermotolerance has been identified in African rice* O. glaberrima* accession CG14. This gene encodes *α*2 subunit of the 26S proteasome, which protects cells from heat stress through more efficient elimination of cytotoxic denatured proteins. This gene was demonstrated to have more effective maintenance of heat-response processes than its counterparts* (OsTT1)* in* O. sativa* [88]. These QTLs may also constitute a genetic reservoir to improve indirectly this trait in rice.

## 4. Genetic Approaches to Reduce Heat Stress Effect on Plant

Zou et al. [89] proposed a combination of transcriptomic, proteomic, and metabolomic approaches to characterize regulation network in response to heat stress in rice. Moreover, stress tolerance in plant was reported to be dependable on expression of heat stress-tolerant genes [90]. Due to the complex mechanism pathways underlying heat stress occurrence in rice, exploitation of different thermotolerant germplasms could be crucial to reduce stress effect. Since there are allelic or nonallelic relations between rice varieties for multiple traits, crossing the rice varieties with nonallelic relationship for heat tolerance could help to gather in one recipient plant different heat tolerance related genes. Pyramiding the nonallelic heat tolerance QTLs is regarded more accurate to alleviate chalkiness problem under heat stress. This suggests that plant thermotolerance ability could be enhanced by breeding efforts, even though it is a complicated and complex trait.

## 5. Perspectives: Pyramiding of Nonallelic Stable QTLs Controlling Low Chalkiness Formation under HT Condition in Rice

**Figure 3:**
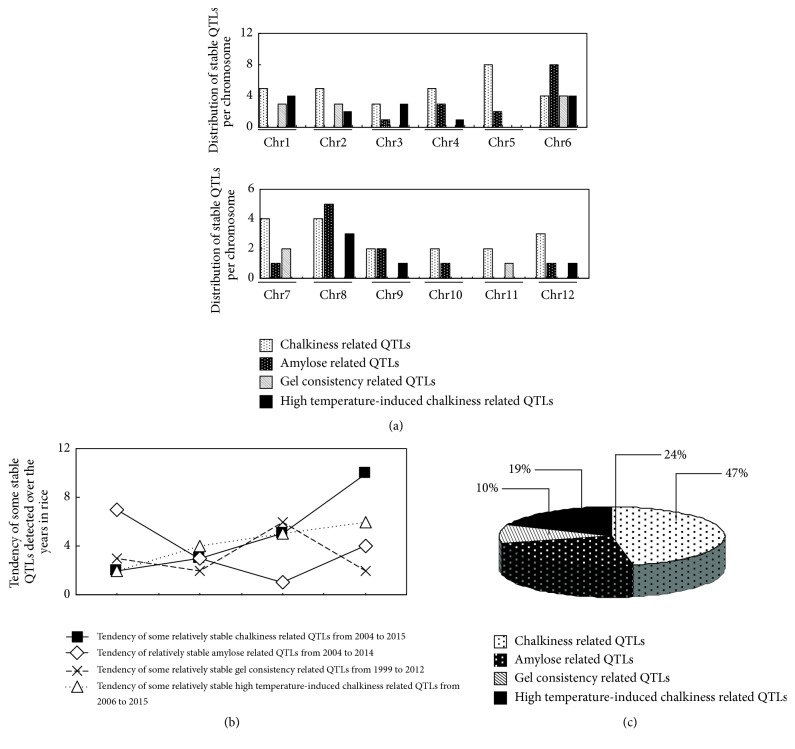
Distribution and evolution of relatively stable QTLs related to chalkiness, amylose content, gel consistency, and high temperature-induced chalkiness QTLs. (a) Distribution of stable quality related QTLs over the 12 rice chromosomes. (b) Evolution of some of the relatively stable QTLs reported in rice. (c) Proportion of each of quality trait related QTLs as compared with the total 4 rice quality related QTLs studied (diagram showing the percentage of each type of QTLs among the 4 stable grain quality QTLs studied).

**Figure 4:**
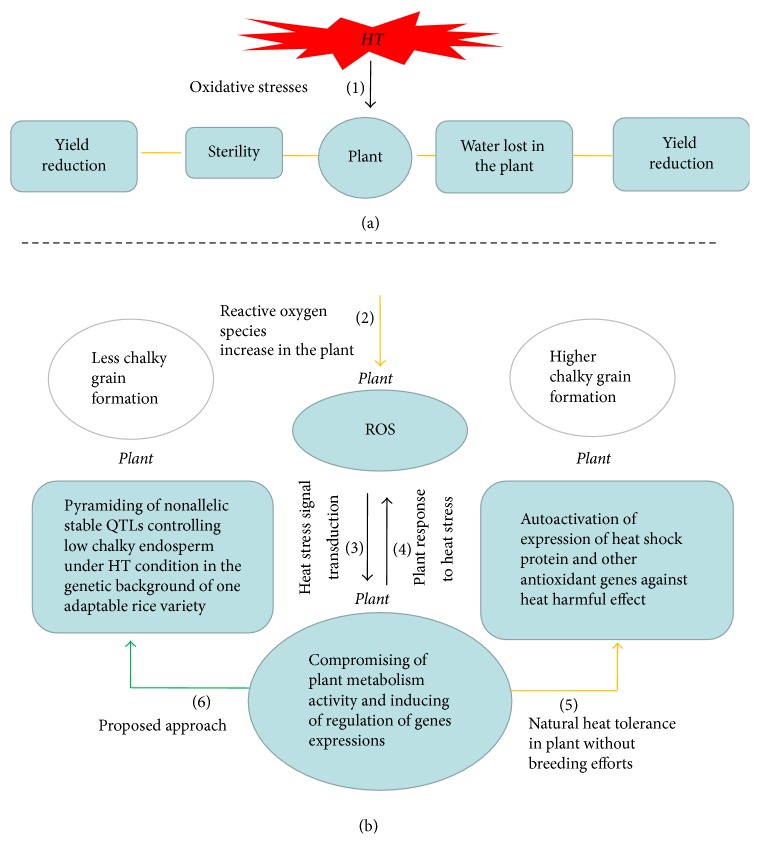
Proposed approach for mitigating heat stress damage in plant. (1) Oxidative stresses from higher temperature. (2) Reactive oxygen species production. (3) Heat stress signal transduction in the plant and change in metabolic activity and genes expression. (4) Plant response to heat stress. (5) Heat stress tolerance via the natural metabolic defense. (6) Defect in HS tolerance and chalkiness formation. Heat stress tolerance via heat tolerance genes pyramiding. Stress tolerance and less chalky grain formation. Heat stress effect on plant fertility and yield reduction. Heat stress on plant water content. Water lost and grain filling rate (GFR) reduction. Effect of GFR on grain yield. Effect of GFR on chalkiness formation in rice. Yellow lines indicated detrimental effect of HS on the rice plant with weak tolerance. Green lines showed tolerance for the HS from strengthened metabolic defense. Black lines indicate the detrimental action of ROS and transcriptional responsiveness of the plant.

It is clear that the gradual increase of the global warming affects not only grain yield but also the quality of rice. It is worth noting that an increase of temperature during the growing season of rice can accelerate the formation of the chalkiness phenomenon. Numerous studies have shed light on the genetic mechanism that regulates the heat stress response in rice. In general, many cultivated rice varieties were selected as potential donor parents of stress-tolerant QTLs (Asian rice varieties such as Kasalath, Chikushi 52, Hanaechizen, and Koshijiwase and African rice accession CG14). However, up to date, no rice variety was certified to be completely tolerant to an increasing world temperature, so breeders are still concerned about the heat stress issue in rice production. Detection and insertion of de novo heat tolerance gene into a given rice germplasm could somewhat alleviate heat stress effects on the endosperm but not truly possible definitely due to their complex metabolism pathways. Chalkiness occurrence depends on multiple genes, of which expression pathways are poorly understood. The expressions of chalkiness related genes were described to be affected under heat stress condition. Meanwhile, some heat-responsive genes and the QTLs related to starch metabolism, protein storage, and chalkiness were documented to be colocated in rice genome assets of QTLs. Expression of heat stress-responsive genes increases, while that of starch metabolism and protein storage decreased under high-temperature condition as highlighted in Figure 1(b). We speculated that this opposite expression pattern might be coordinated by an upstream regulator, which acts in heat response and carbohydrates/protein synthesis pathways under high-temperature condition. Otherwise, this may be due to genetic interactions (gene-gene, gene-protein, and protein-protein) between high-temperature responsive genes and QTLs related to starch metabolism, protein storage, and chalkiness formation. It appears that the disturbance of metabolic function begins when internal plant temperature exceeds 27°C (Figure 2). However, more studies will be needed to verify and clarify this statement. These sets of QTLs, including heat-responsive QTLs, starch-metabolizing QTLs, and chalkiness related QTLs, were distributed throughout rice genome [27]. For this reason, modifying expression of a single QTL is unlikely to modify a whole regulatory mechanism that is involved in chalkiness formation. It is understandable that the collection and combination of different nonallelic heat-tolerant QTLs would be a prerequisite for restoring the abnormal genetic interactions between heat stress-responsive and starch-metabolizing QTLs. In this review, the detection of chalkiness and HT induced chalkiness related QTLs is demonstrated to be increasing over the time, while the evolution of the amylose content and gel consistency related QTLs is slowed, meaning that chalkiness problem is very important and still remains incompletely solved. In this review, chromosome covering analysis of some stable HT induced chalkiness related QTLs is an example of the hope for finding new QTLs given that some chromosomes (5, 7, 10, and 11) are not yet fully explored and exploited (Figure 3). However, this requires the collection and screening of different rice germplasm without neglecting the available HT-tolerant varieties. This review demonstrates the possibility to still identify novel QTLs, which can be useful in alleviating the chalkiness problem. Therefore, we propose a pragmatic genetic approach like assembly of germplasm with considerable heat tolerance ability and the pyramiding of these QTLs into adaptable rice varieties (Figure 4). The current approach involves exploiting the synergic heat tolerance effects of these sets of QTLs. Richard [91] and Hasanuzzaman et al. [92] reported that gene pyramiding could improve traits whose controlling gene has been identified or not identified. Gene pyramiding via marker-assisted selection can be proposed as a potentially good method due to its effectiveness to combat the chalkiness issue.

Reduction in percentage of chalkiness can ensure production of rice grains with good quality. This review presented genetic and enzymatic factors that are associated with chalkiness formation. Moreover, the colocalization of high-temperature-responsive QTLs and that of chalkiness and starch metabolism offer explanations for deterioration of the grain endosperm under heat stress condition. For this reason, we propose pyramiding of stable nonallelic thermoresistant QTLs associated with low chalky rate into one rice variety as a solution to reduce chalky grain formation rates in rice. Furthermore, we suggest subsequent research studies to be conducted which can unlock knowledge to better understand genetic interactions that exist between high-temperature-responsive QTLs and those controlling chalkiness or starch metabolism.

## Figures and Tables

**Table 1:** Some major effect and relatively stable QTLs associated with chalkiness trait in rice.

Type of chalkiness	QTLs	Chr^x^	Associated markers	Physical map position (Mb)	Parents	Population type	LOD	Add effect	References
Chalkiness	*qPGWC.NH-1.1*	1	RM7124	24.38	Sasanishiki/Habataki	ILs	4.965	0.22	Bian et al. (2014)
Chalkiness	*qPGWC.NH-1.2*	1	RM7600	39.16	Sasanishiki/Habataki	ILs	4.546	0.21	Bian et al. (2014)
Chalkiness	*qPGWC.NC-2*	2	RM5699	8.98	Sasanishiki/Habataki	ILs	4.211	0.17	Bian et al. (2014)
Chalkiness	*qPGWC.NC-11*	11	RM1341	20.80	Sasanishiki/Habataki	ILs	4.119	0.17	Bian et al. (2014)
Chalkiness	*qPGWC.NC-4*	4	RM7585	0.21	Sasanishiki/Habataki	ILs	3.929	0.17	Bian et al. (2014)
Chalkiness	*qPGWC.HN-9*	9	P414D03	d	Sasanishiki/Habataki	ILs	4.198	0.12	Bian et al. (2014)
Chalkiness	*qPGWC-5*	5	M413–RM169	^ ^d	“N22”/“Nanjing35”	F_2_	2.75	−10.37	B. Y. Lu et al. (2013)
Chalkiness	*qPGWC-6*	6	R2869-R1952	d	Kasalath/Koshihikari	CSSL	8.13	14.77	Zheng et al. (2012)
Chalkiness	*qPGWC-2*	2	C1419-C560	d	Kasalath/Koshihikari	CSSL	2.68	12.91	Zheng et al. (2012)
Chalkiness	*qPGWC-3*	3	S879-S1513	d	Kasalath/Koshihikari	CSSL	3.01	8.14	Zheng et al. (2012)
Chalkiness	*qCR2*	2	RM145-RM3284	4.86–18.55	*O. sativa/O. rufipogon*	ILs	6.7	−14.6	P. R. Yuan et al. (2010)
Chalkiness	*qPGWC-7*	7	RM234	17.50	PA64s/9311	CSSL	27.75	22.63	Zhou et al. (2009)
Chalkiness	*qPCRG-8 (qDC-8)*	8	SA1656-RA2676	d	Koshihikari/Nonabokra	CSSL	NA	−1.96	Hao et al. (2009)
White-back	*qWB*3^b^	3	RM4512	11.28	Hanaechizen/Niigatawase	RILs	4.60	0.73	Kobayashi et al. (2007)
White-back	*qWB*4^b^	4	RM3288	27.51	Hanaechizen/Niigatawase	RILs	4.36	0.53	Kobayashi et al. (2007)
White-back	*qWB*6^b^	6	RM3034	42.57	Hanaechizen/Niigatawase	RILs	13.39	1.14	Kobayashi et al. (2007)
White-back/basal-white	*qAPG5-1*	5	C10987-RM6645	12.99–15.00^c^	Koshihikari/Kasalath	NA	NA	NA	Ebitani et al. (2006)
White-back/basal-white	*qAPG5-2*	5	E10886-RM3476	20.13–23.82^c^	Koshihikari/Kasalath	NA	NA	NA	Ebitani et al. (2006)
Basal-white	*qBW*2^b^	2	RM3294	5.20	Koshihikari/Kasalath	NA	12.4	9.6	Ebitani et al. (2005)
Basal-white	*qBW1*2^b^	12	RM1208	1.07^c^	Koshihikari/Kasalath	NA	4.1	7.1	Ebitani et al. (2005)
Chalkiness	*qPGWC-8*	8	G1149-R727	19.17–26.23^c^	Asominori/IR24	CSSLs	5.2–7.3	21.8–33.7	Wan et al. (2005)
Chalkiness	*qPGWC-9*	9	XNpb36-XNpb103	1.81–10.8^c^	Asominori/IR24	CSSLs	3.0–5.0	16.8–33.8	Wan et al. (2005)
White-core	*wc12.1*	12	RZ397	5.76^c^	V20A/IRGC 103544	BC_3_F_1_	3.8	9.87	Li et al. (2004)
White-core	*wca3.1*	3	RM148	35.78^c^	V20A/IRGC 103544	BC_3_F_1_	4.5	14.79	Li et al. (2004)
Chalkiness	*qCH*1^b^	1	C161-R753	0.83^c^	Zhenshan 97/Minghui 63	F_2:3_	2.6	1.97	Tan et al. (2000)
Chalkiness	*qCH5-*1^b^	5	RG360-C734	7.78^c^	Zhenshan 97/Minghui 63	F_2:3_	29.3	30.91	Tan et al. (2000)
Chalkiness	*qCH5-*2^b^	5	RG528-C1447	27.86^c^	Zhenshan 97/Minghui 63	F_2:3_	5.8	13.74	Tan et al. (2000)
Chalkiness	*qCH*6^b^	6	R1952-C226	2.90^c^	Zhenshan 97/Minghui 63	F_2:3_	2.5	8.24	Tan et al. (2000)
Chalkiness	*qCH1*0^b^	10	R2625-C223	21.78^c^	Zhenshan 97/Minghui 63	F_2:3_	2.5	8.57	Tan et al. (2000)
White-belly	*qWB5*s^b^	5	RG360-C734	10.65^c^	Zhenshan 97/Minghui 63	F_2:3_	35.2	72.9	Tan et al. (2000)
White-belly	*qWB7*s^b^	7	R1245-R1789	25.86^c^	Zhenshan 97/Minghui 63	F_2:3_	2.7	24.5	Tan et al. (2000)
White-core	*qWC*5^b^	5	RG360-C734	14.26^c^	Zhenshan 97/Minghui 63	F_2:3_	4.5	12.2	Tan et al. (2000)
White-core	*qWC*6^b^	6	Wx-R1952	1.77^c^	Zhenshan 97/Minghui 63	F_2:3_	4.0	9.8	Tan et al. (2000)
Chalkiness	*qDEC*4^s^	4	id4007289-id4008855	22.01–25.15	Lemont/Teqing	RILs	NA	2.8	X. Zhao et al. (2015)
Chalkiness	*qPGWC*4^s^	4	id4007289-id4008855	22.01–25.15	Lemont/Teqing	RILs	NA	9.2	X. Zhao et al. (2015)
Chalkiness	*qPGWC*4^s^	4	id4007289-id4008855	22.01–25.15	Lemont/Teqing	RILs	NA	11.1	X. Zhao et al. (2015)
Chalkiness	*qGL7.*1^s^	7	S07_22019132	3.51*E* − 05	Using GWAS	272 rice varieties	NA	−1.04	X. Qiu et al. (2015)
Chalkiness	*qGW11.*1^s^	11	D11_7124485	8.76*E* − 05	Using GWAS	272 rice varieties	NA	0.12	X. Qiu et al. (2015)
Chalkiness	*qTGW2.*1^s^	2	S02_30688426	8.86*E* − 05	Using GWAS	272 rice varieties	NA	1.78	X. Qiu et al. (2015)
Chalkiness	*qBRR2.*2^s^	2	D02_25652984	1.96*E* − 05	Using GWAS	272 rice varieties	NA	−4.75	X. Qiu et al. (2015)
Chalkiness	*qDEC1.*1^s^	1	S01_5811836	8.66*E* − 05	Using GWAS	272 rice varieties	NA	11.18	X. Qiu et al. (2015)
Chalkiness	*PGC1*0^s^	10	68923-PGC	NA	Zhenshan 97A/Minghui 63	RILs	NA	NA	X. Liu et al. (2015)
Chalkiness	*qPGC8–*2^s^	8	Bin 393	4.6–26.1	Nipponbare/Zhenshan 97A	CSSL	NA	0.045	W. Sun et al. (2015)
White-belly rate	*qWBR*1^s^	1	RM490-RM600	9.4	Zhenshan 97A/Nanyangzhan	RILs	3.3	12.61	B. Peng et al. (2014)
White-belly rate	*qWBR*8^s^	8	RM264-RM477	28.1	Zhenshan 97A/Nanyangzhan	RILs	3.0	11.05	B. Peng et al. (2014)
White-belly rate	*qWBR1*2^s^	12	RM101-RM519	20	Zhenshan 97A/Nanyangzhan	RILs	3.6	14.55	B. Peng et al. (2014)
Chalkiness rate	*qCR*5^s^	5	MRG5972-RM480	27.2	Zhenshan 97A/DL208	RILs	3.2	10.5	B. Peng et al. (2014)
White-core rate	*qWCR*7^s^	7	RM445-RM418	18.1	Zhenshan 97A/DL208	RILs	2.7	−6.78	B. Peng et al. (2014)

x indicates chromosome in abbreviation; b indicates QTLs named tentatively by Yamakawa et al. (2008) in their report;c indicates physical map position calculated by Yamakawa et al. (2008) in their report; d indicates insufficiency of information to calculate physical map position for the related loci in this report; s indicates relatively stable QTLs detected throughout different growing conditions. NA: data is not available.

**Table 2:** Certain content QTLs associated with amylose content trait in rice.

QTLs	Chr^x^	Associated markers	Parents	Population type	LOD	Add effect	References
*qHAC4(HT)*	4	M13.4-M15.9^z^	9311/Nipponbare	CSSLs	4.93	NA	Zhang et al. (2014)
*qHAC8a(HT)*	8	M0.7-M1^z^	9311/Nipponbare	CSSLs	6.19	NA	Zhang et al. (2014)
*qHAC8b(HT)*	8	M8.7-M21.2^z^	9311/Nipponbare	CSSLs	5.59	NA	Zhang et al. (2014)
*qHAC10(HT)*	10	M19.8-M20.5^z^	9311/Nipponbare	CSSLs	5.20	NA	Zhang et al. (2014)
*qDC-8*	8	RM5911-SA1656	Koshihikari/Nonabokra	CSSLs	NA	−0.05	Hao et al. (2009)
*ac6.1*	6	RM314–RM3	Swarna/IRGC81848	BC_2_F_2_	2.60	0.86	Swamy et al. (2012)
*qPCRG-6(qDC-6)*	6	RA1952-RA2349	Koshihikari/Nonabokra	CSSLs	NA	5.19	Hao et al. (2009)
*qDC-4*	4	R1854-RA0288	Koshihikari/Nonabokra	CSSLs	NA	−0.02	Hao et al. (2009)
*qAC-8*	8	G1149–R727	Asominori and IR25	CSSLs	3.70	1.00	X. Y. Wan et al. (2004)
*qAC-9a*	9	XNpb36–XNpb103	Asominori and IR26	CSSLs	2.50	1.40	X. Y. Wan et al. (2004)
*qAC-9b*	9	C609–C506	Asominori and IR27	CSSLs	3.00	0.80	X. Y. Wan et al. (2004)
*qAC-12*	12	XNpb189-2–XNpb24-2	Asominori and IR28	CSSLs	2.30	0.80	X. Y. Wan (2004)
*amy8*	8	RM230–RM264	Caiapo/IRGC 103544	DHs	3.10	−1.85	G. Aluko (2004)
*amy6*	6	RM190–RM253	Caiapo/IRGC 103544	DHs	19.30	−2.60	G. Aluko (2004)
*amy3*	3	RM7–RM251	Caiapo/IRGC 103544	DHs	3.70	−2.73	G. Aluko (2004)
*qAC-6*	6	R2869-R1962	Nipponbare/Kasalath	BILs	35.60	−5.28	Li et al. (2003)
*qAC-6*	6	R2869-R1962	Nipponbare/Kasalath	BILs	43.50	−4.40	Li et al. (2003)
*qAC-3*	3	R1927-R3226	Nipponbare/Kasalath	BILs	2.50	0.71	Li et al. (2003)
*qAC-4*	4	C1100-R1783	Nipponbare/Kasalath	BILs	3.20	−0.90	Li et al. (2003)
*qAC-5*	5	C624-C128	Nipponbare/Kasalath	BILs	2.80	−0.92	Li et al. (2003)
*qA*C^f^	6	RM170	IR64/IRGC105491	BC_2_F_2_	14.63	−0.88	E. M. Septiningsih et al. (2003)
*qAA*C^f^	7	RG375/RG477	IR64/Azucena	DHs	2.61	0.76	J. S. Bao et al. (2002)
*qW*x^f^	6	R565(F2:3); C952-C1496(RILs)^g^	Zhenshan 97/Minghui 63	F2:3; RILs	69.00	NA	Y. F. Tan et al. (1999)
*qAC-5*	5	RG573-C624	ZYQ8/JX17	DHs	2.67	−3.32	P. He et al. (1999)
*Wx*	6	*Wx*	ZYQ8/JX17	DHs	28.39	−8.52	P. He et al. (1999)

x indicates chromosome in abbreviation; f indicates amylose content QTL named tentatively in this report; g indicates the associated markers reported based on type of population used; z indicates associated markers tentatively named in this report based on the physical map position of the related QTLs. NA: data is not available; HT: QTL detected for amylose content in response to high temperature.

**Table 3:** Certain QTLs associated with gel consistency trait detected in rice.

QTLs	Chr^x^	Associated markers	Parents	Population ype	LOD	Add effect	References
*gc1.1*	1	RM499–RM428	Swarna/IRGC81848	BC_2_F_2_	3.90	5.67	Swamy et al. (2012)
*gc1.2*	1	RM580–RM81	Swarna/IRGC81848	BC_2_F_2_	3.20	6.45	Swamy et al. (2012)
*gc11.1*	11	RM209–RM21	Swarna/IRGC81848	BC_2_F_2_	2.90	4.25	Swamy et al. (2012)
*qGC-1*	1	C122-R886	Nipponbare/Kasalath	BILs	3.00	5.34	Li et al. (2003)
*qGC-2a*	2	R712-G227	Nipponbare/Kasalath	BILs	4.00	6.11	Li et al. (2003)
*qGC-2b*	2	G1314B-G132	Nipponbare/Kasalath	BILs	2.20	4.47	Li et al. (2003)
*qGC-6a*	6	L688-G200	Nipponbare/Kasalath	BILs	4.60	−7.65	Li et al. (2003)
*qGC-6b*	6	C556-R2071	Nipponbare/Kasalath	BILs	2.50	−4.99	Li et al. (2003)
*qG*C^f^	6	RM50	IR64/IRGC 105491	BC_2_F_2_	4.02	3.57	Septiningsih et al. (2003)
*qGC-*1^f^	1	RG331/RG810	IR64/Azucena	DHs	2.71	−3.70	Bao et al. (2002)
*qGC-*7^f^	7	RG477/PGMSO.7	IR64/Azucena	DHs	3.54	−4.65	Bao et al. (2002)
*qG*C^f^	6	R565(F_2:3_); C952-C1496(RILs)^g^	Zhenshan 97/Minghui 63	F_2:3_; RILs	57.00	NA	Tan et al. (1999)
*qGC-2*	2	RG171-G243A	ZYQ8/JX17	DHs	4.14	17.60	He et al. (1999)
*qGC-7*	7	TCT122-RG769	ZYQ8/JX17	DHs	3.26	17.00	He et al. (1999)

x indicates chromosome in abbreviation; f indicates gel consistency QTL named tentatively in this report; g indicates the associated markers reported based on type of population used. NA: data is not available.

**Table 4:** Certain white-back chalkiness related QTLs detected under heat stress condition in rice.

QTLs	Chr^x^	Associated markers	Physical map position (Mb)	Parents^d^	Heat treatment condition	Population type	LOD	Add effect	References
*qWB1*	1	RM7075	15.10	Tsukushiroman/Chikushi 52	>27°C (irrigated with hot water, 35°C)	RILs	4.02	–3.82	Takuya et al. (2015)
*qWB8*	8	RM72	18.75	Tsukushiroman/Chikushi 52	>27°C (irrigated with hot water, 35°C)	RILs	4.34	–1.44	Takuya et al. (2015)
*qBW2*	2	RM5470	27.17	Tsukushiroman/Chikushi 52	>27°C (irrigated with hot water, 35°C)	RILs	2.93	–1.17	Takuya et al. (2015)
*qBW6*	6	RM20429	25.04	Tsukushiroman/Chikushi 52	>27°C (irrigated with hot water, 35°C)	RILs	2.78	1.40	Takuya et al. (2015)
*qBW12*	12	RM1986	21.28	Tsukushiroman/Chikushi 52	>27°C (irrigated with hot water, 35°C)	RILs	3.35	1.60	Takuya et al. (2015)
*qWB3*	3	RM4853	1.37	Tsukushiroman/Chikushi 52	>27°C (irrigated with hot water, 35°C)	RILs	8.90	–8.39	Takuya et al. (2015)
*qWB8*	8	RM3689	19.33	Tsukushiroman/Chikushi 52	>27°C (irrigated with hot water, 35°C)	RILs	2.21	–4.45	Takuya et al. (2015)
*qBW3*	3	RM4853	1.37	Tsukushiroman/Chikushi 52	>27°C (irrigated with hot water, 35°C)	RILs	4.59	–1.41	Takuya et al. (2015)
*qWB3*	3	RM4383	14.72	Niigatawase/Hanaechizen	>27°C	RILs	5.18	−3.11	Kobayashi et al. (2007, 2013)
*qWB4*	4	RM3288	27.51	Niigatawase/Hanaechizen	>27°C	RILs	4.30	−2.42	Kobayashi et al. (2007, 2013)
*qWB6*	6	RM8125	3.16	Niigatawase/Hanaechizen	>27°C	RILs	18.11	−5.40	Kobayashi et al. (2007, 2013)
*qWB9*	9	RM2482	22.27	Niigatawase/Hanaechizen	>27°C	RILs	7.63	4.35	Kobayashi et al. (2007, 2013)
*qGH*6^b^	6	RM3034	4.25	Niigatawase/Hanaechizen	>27°C	RILs	7.80	0.19	Kobayashi et al. (2007, 2013)
*qWK1-1*	1	RM8068	1.65	Chiyonishiki/Koshijiwase	25–27°C at night/30–32°C at daylight	RILs	3.4	8.9	Tabata et al. (2007)
*qWK1-2*	1	RM5501	34.54	Chiyonishiki/Koshijiwase	25–27°C at night/30–32°C at daylight	RILs	5.6	15.0	Tabata et al. (2007)
*qWK2*	2	RM5916	34.08	Chiyonishiki/Koshijiwase	25–27°C at night/30–32°C at daylight	RILs	3.8	9.3	Tabata et al. (2007)
*qWK8*	8	RM2680	0.14	Chiyonishiki/Koshijiwase	25–27°C at night/30–32°C at daylight	RILs	3.6	9.2	Tabata et al. (2007)
*qWBHT*1^b^	1	S13781	35.11^c^	Kokoromachi/Tohoku 168	NA	NA	4.3	4.6	Shirasawa et al. (2006)
*qWBHT*2^b^	6	WxCT	1.77^c^	Tohoku 168/Kokoromachi	NA	NA	8.6	5.6	Shirasawa et al. (2006)

x indicates chromosome in abbreviation; b indicates QTLs named tentatively by Yamakawa et al. (2008) in their report;c indicates physical map position calculated by Yamakawa et al. (2008) in their report; d indicates parent with decreasing chalky grain percentage allele. NA: data is not available.
